# Prostate specific antigen testing in family practice: a cross sectional survey of self-reported rates of and reasons for testing participation and risk disclosure

**DOI:** 10.1186/1471-2296-14-186

**Published:** 2013-12-09

**Authors:** Mariko Carey, Jamie Bryant, Sze Lin Yoong, Grant Russell, Daniel Barker, Rob Sanson-Fisher

**Affiliations:** 1 Priority Research Centre for Health Behaviour, Faculty of Health, University of Newcastle, Callaghan 2308, Australia; 2 Hunter Medical Research Institute, Newcastle 2305, Australia; 3 Southern Academic Primary Care Research Unit, Monash University, Notting Hill 3168, Australia

**Keywords:** Prostate cancer, Screening, Prostate specific antigen, Family practice

## Abstract

**Background:**

Despite controversy about the benefits of routine prostate specific antigen (PSA) testing, rates of participation continue to rise. It is important to ensure that men are fully informed about the potential risks associated with this test. Little is known about the processes of shared decision making for PSA testing in the family practice setting. This study aimed to explore men’s experiences of PSA testing participation and risk disclosure for PSA testing.

**Methods:**

A cross-sectional survey of male family practice attendees aged 40 years or older, with no previous history of prostate cancer, between June 2010 and November 2011. Questions related to whether participants had undertaken PSA testing or discussed this with their doctor over the past 5 years, whether the patient or doctor had initiated the discussion, reasons for undergoing testing, and whether their doctor had discussed particular risks associated with PSA testing.

**Results:**

Sixty-seven percent (215/320) of men recalled having a PSA test in the past five years. Of the respondents who reported not having a test, 14% had discussed it with their doctor. The main reasons for having a PSA test were doctor recommendation and wanting to keep up to date with health tests. Thirty-eight percent or fewer respondents reported being advised of each potential risk.

**Conclusions:**

Despite debate over the benefits of routine PSA testing, a high proportion of male family practice attendees report undertaking this test. Risks associated with testing appear to be poorly disclosed by general practitioners. These results suggest the need to improve the quality of informed consent for PSA testing in the family practice setting.

## Background

Prostate cancer is one of the most common cancers among men world-wide [1]. Elevated levels of prostate specific antigen (PSA) in the blood can indicate the presence of prostate cancer as well as a number of other benign conditions. As such, there has been increased use of PSA testing for the detection of prostate cancer in the population. There is significant variation across countries in rates of PSA testing to detect the disease in its early stages [2]. An American population survey reported that 41% of men aged 50 or older reported having had a PSA test within the past year [3]. Health service use data indicates that approximately 21% of Australian men underwent PSA testing in 2006 [2]; while only 6% of men aged 45–89 in the family practice setting in the United Kingdom undergo testing each year [4,5].

### PSA testing remains controversial

Despite the relatively high rates of PSA testing, routine screening remains controversial [6]. The results of the two largest clinical trials are conflicting about whether routine screening results in decreased mortality [7,8]. A recent meta-analysis concluded that current evidence does not support the use of the PSA test for screening [9]. One recent randomized controlled trial estimated that 1068 men would need to be screened twice during a nine year period to save one life, and 48 men would need to be treated for prostate cancer to save one life [10]. Currently the Urological Society of Australia and New Zealand (USANZ) does not recommend routine PSA testing [11]. However it does recommend PSA testing, with digital rectal examination, be offered to men aged 55–69 years after providing information about the risks and benefits of such testing [11]. The US Preventive Services Task Force does not recommend PSA testing for asymptomatic men regardless of age [12].

### What risks should be disclosed to patients making a decision about PSA testing?

There are a number of risk and benefits with undertaking PSA testing. Potential risks of PSA testing include a high false positive rate (up to 76%), an indicator for biopsy which carries an associated risk of infection, bleeding and pain [13]. Since most men with prostate cancer will die from other causes before their cancer becomes symptomatic, over-diagnosis and over-treatment are significant concerns [6]. Rates of over-diagnosis have been estimated to range from 23% to 42% of all prostate cancer detected through screening [6]. Up to 90% of men with low PSA values receive early intervention [14,15]. Over-treatment for prostate cancer carries a significant risk of adverse effects. Surgical treatment by radical prostatectomy has not been found to reduce mortality compared to no treatment, and is associated with high rates of erectile dysfunction and urinary incontinence [16,17].

### How well are risks disclosed in practice?

Prior research suggests that men’s knowledge of the benefits and risks of PSA testing is poor [18,19]. The 2000 American National Health Interview Survey found that 33% of the 8087 participating men aged 40 or older reported having received a PSA test [20]. However, only 65% of those screened reported discussing the risk and benefits of testing with their doctor. Similarly, an Australian study indicated that only 62% of men screened in the past 5 years recalled a discussion about the risks and benefits of testing [21].

Given that the majority of research has been undertaken in the US, it is unclear how generalizable these findings may be to the experiences of men in family practice settings in other countries. The Australian health care setting, for example, differs substantially to the US, with access to general practitioners (GPs) available at little or no cost to the patient. It is therefore useful to investigate whether associations between high socioeconomic status and increased screening rates identified in previous research are applicable to the Australian setting [4,5,18]. All Australians have access to Medicare, a government funded universal health insurance program that reimburses patients for fees incurred for core clinical services [22]. The vast majority of GP services are directly billed from the provider to the Medicare (and hence delivered at no cost to the patient). Private health insurance underwrites access to both allied health professionals and to private hospital inpatient care [22].

The current study was conducted as part of a large cross sectional study exploring screening participation for cancer and cardiovascular disease among Australian family practice attendees [23]. A subsample of male participants was asked to complete questions related to PSA testing. We aimed to: 1) identify the proportion of male family practice patients who report having a) undergone a PSA test; or b) discussed PSA testing with their doctor, in the last 5 years; 2) identify factors associated with having a test and perceived reasons for having a test; and 3) explore how discussion of PSA testing was initiated and the proportion of patients who were told about risks associated with testing.

## Methods

### Study design and setting

A cross-sectional study, conducted with patients presenting to 12 family practices in Australia between 16th June 2010 and 18th November 2011.

### Recruitment of family practices

Geographic areas within 20 kilometers from a University Department of Family Practice were selected in Newcastle and Sydney. A list of practices was generated using the Medical Directory of Australia and the Yellow Pages telephone directory. In Melbourne, a random list of practices within a region of the city corresponding to the boundaries of a Division of General Practice was generated from a commercially available database, Australasian Medical Publishing Company (AMPCo) [24]. Practices were mailed an invitation to participate in the study which was followed up with one to three telephone calls and, if requested, in-person visits, by a member of the research team. Practices were approached until four in each region consented. Family practices were eligible if a sufficient number of GPs (equal to two, full time equivalent) agreed to take part.

### Participants

Male patients aged 40 or older, without a history of prostate cancer, who were presenting for family practice care; had sufficient English to complete the survey and were able to provide informed consent were presented with questions related to PSA testing.

### Recruitment

Patients were approached by a research assistant while waiting for their family practice appointment. Informed consent to participate in a touchscreen computer survey was obtained from all participants. Participants were given a paper copy of the information sheet and also presented with this information on the touchscreen computer used to administer the survey. Prior to commencing the survey, participants were asked to touch “NEXT” onscreen to indicate that they consented to participating in the study. Consenting patients completed a brief survey prior to their consultation. Patients were able to exit the survey if called into their appointment.

### Measures

The questionnaire was administered using a DELL Latitude XT2 laptop. Patients touched their selected responses using their finger or a stylus.

#### Demographic and medical characteristics

Self-report data were collected on age, gender, education, possession of a Veteran’s Affairs Treatment Entitlement Card or Health Care Card, and private health insurance. Veteran’s Affairs Entitlement Cards entitle veterans, war widows/widowers and dependents to free access to certain health services, while Health Care Cards enable access to subsidized prescription medicines and medical services funded by the Australian Government. Participants were asked whether they had ever been told by a doctor or nurse that they had: high blood pressure, high cholesterol, heart problems, diabetes, kidney disease, depression, cancer, stroke or chronic pain.

#### Discussion of PSA testing

Respondents were asked whether they had discussed PSA testing with their doctor within the last 5 years, and if so, who the discussion was initiated by.

#### Discussion of risk of PSA testing

The following introduction was provided: “An elevated PSA reading may indicate both prostate cancer and benign (non-cancerous) conditions. PSA levels alone do not give enough information to diagnose prostate cancer. However, GPs will take the result of the PSA test into account when deciding whether to check for further signs of cancer. The following question asks you about what your doctor told you the first time you discussed PSA testing.” Respondents were then presented with a series of statements and asked to indicate whether the potential risk described had been discussed.

#### PSA test history

Respondents were asked whether they had a PSA test within the last 5 years.

#### Reasons for undertaking PSA testing

Those who reported having had a PSA test were asked to rank their top three reasons why, from: “My doctor suggested it because of the symptoms I had”, “I was worried because prostate cancer runs in my family”, “I heard about the test on TV, radio or newspaper”, “My partner/family member suggested I get tested”, “I like to keep up to date with all kinds of health tests”.

#### Ethical approval

Human Research Ethics Committee approval was obtained from the University of Newcastle, the University of New South Wales and Monash University.

### Statistical analysis

Categorical measures were summarised using frequencies and percentages. Continuous measures were summarised using means and standard deviations. Simple logistic regression was used to examine whether the following variables were associated with undergoing a PSA test: age category (40–49, 50–59, 60–69, 70 plus), having a health care card or Veteran’s Affairs card (yes/no), education level (high school certificate or below, Technical and Further Education (TAFE) or diploma, university or postgraduate), and number of chronic conditions. Multiple logistic regression was used to examine these relationships together.

## Results

### Practice response rate

Forty-eight practices were invited to participate, with 12 (25%) agreeing. Participating practices had on average 6.8 family physicians per practice and 80% employed at least one nurse. All were located in metropolitan areas.

### Patient response rate

Of the eligible patients approached, 1269 agreed to participate giving a consent rate of 88%. A total of 371 patients were male and aged 40 and above. Of those, 320 (86.3%) completed the questions relevant to PSA testing, 51 patients were called to their GP prior to commencing. There was no significant difference in the proportion of consenters and non-consenters (χ_2(1)_: 0.5211; p-value: 0.470). Of the 320 participants completing the prostate screening questions, most (72%) were aged between 40 and 70. Just over half (51%) had private health insurance. Demographic characteristics of consenting patients are shown in Table 1.

**Table 1:** Characteristics of participants

**Characteristic**	**n (%)**
Age group	
40-49	78 (24.4%)
50-59	75 (23.4%)
60-69	75 (23.4%)
70+	92 (28.8%)
Education	
Primary school	4 (1.5%)
Some high school	25 (9.1%)
Year 10	37 (14%)
Completed high school certificate	34 (12%)
Technical and further education (TAFE) certificate or Diploma*	53 (19%)
University or other tertiary qualifications	84 (31%)
Postgraduate qualifications	25 (9.1%)
Other	12 (4.4%)
New patient	
Veterans affairs card	22 (6.9%)
Health care card	82 (26%)
Private health insurance	162 (51%)
Number of chronic conditions	1.47(1.23)

*TAFE is attended as part of trade education for apprentices.

### Rates of participation in PSA testing or discussion of PSA testing within the past 5 years

Of the 320 respondents, the majority (n = 215, 67%) reported having had a PSA test within the past 5 years. Of the remaining 105, 15 (14%) had discussed PSA testing with their doctor. Ten of these 15 participants reported that they had initiated the discussion of PSA testing with their doctor.

### Factors associated with PSA testing

Logistic regression analyses explored whether there was evidence for an association between socio-demographic or medical history factors and having undergone a PSA test within the last 5 years. Those who reported having a test within the last 5 years were more likely to have a greater number of chronic conditions than those who had not (OR = 1.32, 95% CI 1.07 to 1.63, p = 0.0103) (see Table 2). However, after including the other variables of interest in a multiple logistic regression model neither the number of chronic conditions nor any of the other variables were significantly associated with PSA testing within the last 5 years.

**Table 2:** Factors associated with having undertaken a prostate specific antigen test in the last five years

		***PSA Test in last 5 years***		***Simple regression***		***Multiple regression***	
***Variable***		***No (n = 105)***	***Yes (n = 215)***	***Odds ratio (95% CI)***	***P-value***	***Odds Ratio (95% CI)***	***P-value***
Age group	70+	29 (32%)	63 (68%)		.		.
	40-49	39 (50%)	39 (50%)	0.46 (0.25, 0.86)	0.0150	0.48 (0.22, 1.04)	0.0619
	50-59	18 (24%)	57 (76%)	1.46 (0.73, 2.9)	0.2839	1.28 (0.58, 2.81)	0.5410
	60-69	19 (25%)	56 (75%)	1.36 (0.69, 2.68)	0.3804	1.5 (0.7, 3.25)	0.2983
Health care/benefits card	Yes	26 (32%)	56 (68%)		.		.
	No	79 (33%)	159 (67%)	0.93 (0.55, 1.6)	0.8048	0.92 (0.51, 1.67)	0.7939
Education	HSC or below	43 (38%)	69 (62%)		.		.
	TAFE / Diploma	16 (30%)	37 (70%)	1.44 (0.72, 2.9)	0.3057	1.45 (0.68, 3.12)	0.3352
	University / Post Grad	33 (30%)	76 (70%)	1.44 (0.82, 2.51)	0.2048	1.67 (0.88, 3.17)	0.1199
Private health insurance	Yes	50 (31%)	112 (69%)		.		.
	No	55 (35%)	103 (65%)	0.84 (0.52, 1.33)	0.4525	0.73 (0.41, 1.3)	0.2788
Number of Chronic conditions	Mean (Std)	1.21 (1.131)	1.60 (1.261)	1.32 (1.07, 1.63)	0.0103	1.22 (0.94, 1.58)	0.1295

### Reasons for undergoing PSA testing

**Figure 1:**
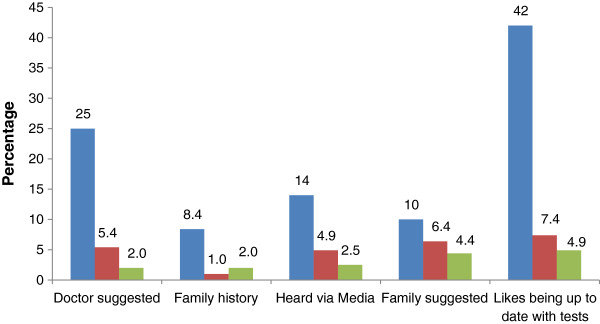
**Reasons for undergoing PSA test.** % 1st ranked reason (blue). % 2nd ranked reason (red). % 3rd ranked reason (green).

Of the 215 men who reported undergoing a test within the past 5 years, 203 also indicated reasons for undertaking testing. Missing data were due to respondents exiting the survey to attend their GP appointment. The top three reasons are reported in Figure 1.

Of the 230 participants who had discussed PSA testing or undergone a test within the last 5 years, 228 indicated how their first discussion of PSA testing with their doctor was initiated. Just over half [n = 123, 54% (95% CI 46.4 to 60.5)] reported that this was doctor initiated, 74 [32%, (95% CI 26.3 to 38.6)] reported that they had initiated the discussion, while 31 [14%, 95% CI 9.1 to 18.1)] could not remember.

### Disclosure of risks regarding PSA testing

Of the 230 respondents who reported having undergone or discussed testing within the last 5 years, 188 answered at least one question related to whether their doctor had provided advice about possible risks. Participants could skip these questions, resulting in some missing data as shown in Table 3. None of the risks were reported to have been disclosed by GPs to more than 38% of respondents. Disclosure of at least one risk was reported by 114 participants (61%). The number and proportion advised of each potential risk are shown in Table 3.

**Table 3:** Number of respondents who report being advised about possible risks of PSA testing

***Received advice about potential risk (n = 188)***	***n (%)***	***95% CI for%***	***Missing values***
No clear evidence that PSA testing saves lives	60 (35%)	(27.5, 41.8)	15
PSA testing can lead to unnecessary treatments	28 (17%)	(11.4, 23.2)	26
Treatment for prostate cancer can lead to incontinence	45 (29%)	(22.0, 36.5)	34
Treatment for prostate cancer can lead to erectile dysfunction	53 (33%)	(25.8, 40.5)	28
Most prostate cancers will not affect a man’s health during his lifetime	65 (38%)	(30.5, 45.1)	16

## Discussion

To our knowledge, this study is one of only a few which have quantitatively examined men’s experiences of PSA testing discussions and reasons for undergoing testing in the Australian family practice setting. Sixty-seven percent of participants reported having had a PSA test in the past five years, and a further 14% discussed it with their doctor. This rate is higher than that reported by Slevin’s 1999 population-based survey in one Australian state [21]. The latter study reported that 43% of men aged 40 to 80 had undergone a PSA test, with most of these (86%) having had their first test within the last 5 years. Gatellari’s study in Australian general practice patients also identified lower rates of testing with only 23.6% of men aged 40–70 years indicating ever having a PSA test and of those, 42% within the last year [25]. Baade’s study reported that 21% of men underwent PSA testing in 2006 [2]; while Arnold-Reed’s study in five family practices indicated that 59% of men aged 40–80 had undergone testing [19]. While this latter finding is closer to the present findings, the results are not directly comparable as no time-frame for testing was used. Methodological differences between studies may account for these discrepancies. Slevin’s data was based on a survey of randomly selected households, while our sample consisted of men sampled from family practices. It is possible that men recruited at a family practice are more health conscious and thus more likely to undergo testing than those in the general population. A second possibility is that rates of testing uptake have increased over time, resulting in a higher reported rate of testing in the present study compared to the Slevin, Gattelari and Arnold-Reed studies [19,21,25]. Baade’s study was based on health service use data over a 12 month period, and so it is not possible to directly compare the current findings with the 21% testing rate they reported [2].

### Factors associated with screening participation

None of the socio-demographic characteristics examined in the present study were found to be associated with PSA testing. However, we found a trend that suggested testing participation rates may be lower among men aged 40–49 compared with older men. A previous Australian study found that age was positively associated with having had a PSA test [25]. However, as this association was not explored within the last 5 years, results are not directly comparable to the current study. A recent Dutch study also found that men with chronic conditions had higher rates of PSA testing [26], however we found no association between chronic conditions and PSA testing in the last 5 years. Differences in these findings are likely explained by differences in the time points at which chronic conditions and PSA testing were assessed. Previous research has also indicated that physician characteristics and attitude towards PSA testing may also be associated with testing uptake [25,27]. It was not possible to link patient responses to data on physician characteristics in the current study, however, this could be explored in future work.

### Reasons for participating in PSA testing

Of men who had undergone PSA testing, the most common top ranked reason was wanting to keep ‘up to date’ with health care testing (42%), followed by doctor recommendation to be tested based on symptoms (25%). Previous Australian research found media publicity (46%) and doctor recommendation (42%) were the two main reasons for undertaking PSA testing [21]. Therefore, the current findings may reflect increased awareness of prostate cancer testing as a result of past media campaigns. These results highlight the influence of GP recommendation, patient perceptions of the need for prostate testing, and the influence of the media, in deciding to undertake testing. The fact that our findings suggest that GPs initiate discussions for at least half of PSA tests performed, but fail to adequately disclose the risk of testing, supports the need for improvements in physician’s communication with patients regarding the appropriateness of PSA testing.

### How well are potential risks disclosed?

Our findings show most respondents indicated that discussion of PSA testing was initiated by their doctor (54%) and that 61% of those who had either discussed or undertaken a test in the past 5 years reported having been advised about one or more risk relating to PSA testing. Rates of discussion of specific risks varied from 17% to 38% with the risk of PSA testing leading to unnecessary treatments having the lowest rate of disclosure. Previous findings in this area are mixed. With regards to patient-reported, doctor-initiated discussions of PSA testing, studies in Australia have indicated the occurrence of such discussions range from 25%- 41% [28,29], yet studies in the US have suggested higher rates (74%) [20]. Similarly, reported discussion of potential risks are varied with a recent study showing as few as 11% of patients recalled being advised of potential risks [28], while other studies are in accordance with our findings [20,21]. A US study of 304 men attending general internal medicine outpatient clinics found that men knew about 50% of the prostate cancer facts assessed [18]. Results are not, however, directly comparable due to differences in the recruitment setting and differences in the questions pertaining to knowledge of risks associated with testing.

### Practice implications

The present study indicates that a high proportion of men in the Australian family practice setting undergo PSA testing. While it was not possible to determine what proportion of PSA testing would be considered clinically appropriate in the current study, only 25% of men reported the investigation of symptoms as one of the main reasons for undergoing testing. However, based on our data it is was not possible to draw conclusions about the proportion of men who underwent testing who were asymptomatic. Given potential difficulties in accuracy of recall of symptoms over a 5 year period, it would be useful for prospective research to examine the relationship between symptom experience and the decision to undertake a PSA test. It is important to identify rates of inappropriate testing as this may have implications in terms of over-diagnosis and treatment resulting in harms to the patient [13], as well as inflation of health-care costs.

Given high rates of testing uptake, it seems critical to improve the rate at which key information about the risks and benefits of PSA testing are discussed. Barriers to such discussions may include patient or physician attitudes or knowledge, and perceived lack of time for discussions by doctors [30-32]. Further research is needed to elucidate which factors may be most important to facilitating informed decision making for PSA testing, and testing interventions targeted toward these.

### Limitations

It is possible that the participating practices were not representative of the broader family practice setting. Comparison with data from practices participating in the national Bettering the Evaluation and Care of Health (BEACH) study, involving over 1000 GPs, however, revealed that practices in the current study were similar to those in BEACH in terms of the number of GPs employed [33]. It is also possible that the men who participated in the study were more health conscious or health literate than those who did not consent. Given the controversy over whether PSA testing is beneficial, it is unclear if such differences would have led to under or over-estimation of rates of PSA testing.

Verification of self-reported screening behavior with medical records was beyond the scope of the present study. A previous study has reported an accuracy rate of 74% for self-reported prostate screening among family practice patients [34]; while a meta-analysis reported a sensitivity of 71% and a specificity of 73% [35]. This suggests that there is likely to be some degree of inaccuracy in the self-report data obtained in the present study, our data nevertheless provides a useful indication of the men’s experiences of PSA testing and discussions within the family practice setting.

## Conclusions

Despite debate over the benefits of routine PSA testing, a high proportion of male family practice attendees report participating in PSA testing. Men report that GP recommendations for screening are influential in the decision to be tested, however, overall risks associated with testing appear to be poorly disclosed by GPs. These results suggest the need to improve the quality of information provision about PSA testing in the family practice setting.

## Competing interests

The authors have no competing interests.

## Authors’ contributions

MC, RSF, GR and SLY conceptualized the study, interpreted results and drafted the manuscript. DB analyzed the data, interpreted results and drafted the manuscript. JB interpreted results and drafted the manuscript. All authors approved the final version of the manuscript.

## Pre-publication history

The pre-publication history for this paper can be accessed here:

http://www.biomedcentral.com/1471-2296/14/186/prepub

## References

[B1] Ferlay J Shin H Bray F Forman D Mathers C Parkin D Cancer Incidence and Mortality Worldwide: IARC CancerBase No. 10 [Internet] GLOBOCAN v12 2008 Lyon, France: International Agency for Research on Cancer

[B2] Baade PD Youlden DR Krnjacki LJ International epidemiology of prostate cancer: geographical distribution and secular trends Mol Nutr Food Res 2009 14 2 171 184 10.1002/mnfr.200700511 19101947

[B3] Swan J Breen N Coates RJ Rimer BK Lee NC Progress in cancer screening practices in the United States: results from the 2000 National Health Interview Survey Cancer 2003 14 6 1528 1540 10.1002/cncr.11208 12627518

[B4] Williams N Hughes LJ Turner EL Donovan JL Hamdy FC Neal DE Martin RM Metcalfe C Prostate-specific antigen testing rates remain low in UK general practice: a cross-sectional study in six English cities BJU Int 2011 14 9 1402 1408 10.1111/j.1464-410X.2011.10163.x 21481132

[B5] Melia J Moss S Johns L Rates of prostate-specific antigen testing in general practice in England and Wales in asymptomatic and symptomatic patients: a cross-sectional study BJU Int 2004 14 1 51 56 10.1111/j.1464-4096.2004.04832.x 15217430

[B6] Wolf AMD Wender RC Etzioni RB Thompson IM D'Amico AV Volk RJ Brooks DD Dash C Guessous I Andrews K American cancer society guideline for the early detection of prostate cancer: update 2010 CA-Cancer J Clin 2010 14 2 70 98 10.3322/caac.20066 20200110

[B7] Andriole GL Crawford ED Grubb RL Buys SS Chia D Church TR Fouad MN Isaacs C Kvale PA Reding DJ Prostate cancer screening in the randomized Prostate, Lung, Colorectal, and Ovarian Cancer Screening Trial: mortality results after 13 years of follow-up J Natl Cancer Inst 2012 14 2 125 132 10.1093/jnci/djr500 22228146 PMC3260132

[B8] Schröder FH Hugosson J Roobol MJ Tammela TLJ Ciatto S Nelen V Kwiatkowski M Lujan M Lilja H Zappa M Prostate-cancer mortality at 11 years of follow-up N Engl J Med 2012 14 11 981 990 10.1056/NEJMoa1113135 22417251 PMC6027585

[B9] Djulbegovic M Beyth RJ Neuberger MM Stoffs TL Vieweg J Djulbegovic B Dahm P Screening for prostate cancer: systematic review and meta-analysis of randomised controlled trials BMJ 2010 14 c4543 10.1136/bmj.c4543 20843937 PMC2939952

[B10] Smith RA Cokkinides V Brooks D Saslow D Shah M Brawley OW Cancer screening in the United States, 2011 CA-Cancer J Clin 2011 14 1 8 30 10.3322/caac.20096 21205832

[B11] Urological Society of Australia and New Zealand Urological Society of Australia and New Zealand PSA Testing Policy 2009 [online]. Available: http://www.usanz.org.au/uploads/29168/ufiles/USANZ_2009_PSA_Testing_Policy_Final1.pdf [Accessed 19 June 2012]

[B12] Moyer VA Screening for prostate cancer: U.S. preventive services task force recommendation statement Ann Intern Med 2012 14 2 120 134 10.7326/0003-4819-157-2-201207170-00459 22801674

[B13] Ilic D O'Connor D Green S Wilt T Screening for prostate cancer Cochrane Database Syst Rev 2006 14 CD004720 16856057 10.1002/14651858.CD004720.pub2

[B14] Cooperberg MR Broering JM Carroll PR Time trends and local variation in primary treatment of localized prostate cancer J Clin Oncol 2010 14 7 1117 1123 10.1200/JCO.2009.26.0133 20124165 PMC2834465

[B15] Welch HG Albertsen PC Prostate cancer diagnosis and treatment after the introduction of prostate-specific antigen screening: 1986–2005 J Natl Cancer Inst 2009 14 19 1325 1329 10.1093/jnci/djp278 19720969 PMC2758309

[B16] Wilt TJ Brawer MK Jones KM Barry MJ Aronson WJ Fox S Gingrich JR Wei JT Gilhooly P Grob BM Radical prostatectomy versus observation for localized prostate cancer N Engl J Med 2012 14 3 203 213 10.1056/NEJMoa1113162 22808955 PMC3429335

[B17] Wilbur J Prostate cancer screening: the continuing controversy Am Fam Physician 2008 14 12 1377 1384 19119557

[B18] Chan ECY Vernon SW O'Donnell FT Ahn C Greisinger A Aga DW Informed consent for cancer screening with prostate-specific antigen: How well are men getting the message? Am J Public Health 2003 14 5 779 10.2105/AJPH.93.5.779 12721144 PMC1447839

[B19] Arnold-Reed DE Hince DA Bulsara MK Ngo H Eaton M Wright AR Jones FR Kaczmarczyk W Marangou AG Brett TD Knowledge and attitudes of men about prostate cancer Med J Aust 2008 14 6 312 314 18803533 10.5694/j.1326-5377.2008.tb02047.x

[B20] Han PKJ Coates RJ Uhler RJ Breen N Decision making in prostate-specific antigen screening: national health interview survey, 2000 Am J Prev Med 2006 14 5 394 404 10.1016/j.amepre.2005.12.006 16627127

[B21] Slevin TJ Donnelly N Clarkson JP English DR Ward JE Prostate cancer testing: behaviour, motivation and attitudes among Western Australian men Med J Aust 1999 14 4 185 188 10494233 10.5694/j.1326-5377.1999.tb123594.x

[B22] Department of Health and Aged Care The Australian Health Care System: An Outline 2000 Canberra: Commonwealth of Australia

[B23] Yoong SL Carey ML Sanson-Fisher RW Russell G Mazza D Makeham M Paul CL Inder KJ D'Este C Touch screen computer health assessment in Australian general practice patients: a cross-sectional study protocol BMJ Open 2012 14 4 10.1136/bmjopen-2012-001405 PMC3448137 22761290

[B24] AMPCo Australiasian Medical Publishing Company 2012 [online]. Available: http://www.ampco.com.au/ [Accessed 14 May]

[B25] Gattellari M Young JM Ward JE GP and patient predictors of PSA screening in Australian general practice Fam Pract 2003 14 3 294 303 10.1093/fampra/cmg311 12738699

[B26] Hamoen EH Reukers DF Numans ME Barentsz JO Witjes JA Rovers MM Discrepancies between guidelines and clinical practice regarding prostate-specific antigen testing Fam Pract 2013 14 6 648 654 10.1093/fampra/cmt045 24107269

[B27] Edlefsen KL Mandelson MT McIntosh MW Andersen MR Wagner EH Urban N Prostate-specific antigen for prostate cancer screening: do physician characteristics affect its use? Am J Prev Med 1999 14 1 87 90 10.1016/S0749-3797(99)00041-0 10429758

[B28] McDowell ME Occhipinti S Gardiner RA Chambers SK Patterns of prostate‒specific antigen (PSA) testing in Australian men: the influence of family history BJU Int 2012 14 3 64 70 22458497 10.1111/j.1464-410X.2012.11050.x

[B29] Pinnock CB Weller DP Marshall VR Self-reported prevalence of prostate-specific antigen testing in South Australia: a community study Med J Aust 1998 14 1 25 28 9695698

[B30] Dunn AS Shridharani KV Lou W Bernstein J Horowitz CR Physician–patient discussions of controversial cancer screening tests Am J Prev Med 2001 14 2 130 134 10.1016/S0749-3797(00)00288-9 11165455 PMC4848038

[B31] Guerra C Jacobs S Holmes J Shea J Are physicians discussing prostate cancer screening with their patients and why or why not? A pilot study J Gen Intern Med 2007 14 7 901 907 10.1007/s11606-007-0142-3 17549576 PMC2219711

[B32] Guerra C Schwartz J Armstrong K Brown J Halbert C Shea J Barriers of and facilitators to physician recommendation of colorectal cancer screening J Gen Intern Med 2007 14 12 1681 1688 10.1007/s11606-007-0396-9 17939007 PMC2219836

[B33] Britt H Charles J Henderson J Bayram C Pan Y Valenti L Harrison C O’Halloran J Fahridin S General practice activity in Australia 2009–10. General Practice series no. 27 2010 Canberra: AIHW

[B34] Jordan TR Price JH King KA Masyk T Bedell AW The validity of male patients' self-reports regarding prostate cancer screening Prev Med 1999 14 3 297 303 10.1006/pmed.1998.0430 10072749

[B35] Rauscher GH Johnson TP Cho YI Walk JA Accuracy of self-reported cancer-screening histories: a meta-analysis Cancer Epidemiol Biomarkers Prev 2008 14 4 748 757 10.1158/1055-9965.EPI-07-2629 18381468

